# “Chronic granulomatous invasive fungal rhinosinusitis associated with SARS-CoV-2 infection: A case report”

**DOI:** 10.1016/j.amsu.2021.103129

**Published:** 2021-12-02

**Authors:** Jose Luis Treviño-Gonzalez, Karla Marisol Santos-Santillana, Felix Maldonado-Chapa, Josefina Alejandra Morales-Del Angel, Paola Gomez-Castillo, Jose Rosmal Cortes-Ponce

**Affiliations:** Otolaryngology and Head and Neck Surgery Division, School of Medicine and University Hospital “Dr. Jose E. González”, Universidad Autónoma de Nuevo León, Dr. José Eleuterio González (Gonzalitos) S/N, Mitras Centro, 64460, Monterrey, N.L., Mexico

**Keywords:** Invasive fungal infections, Mycoses, Sinusitis, Paranasal sinus diseases, Aspergillus

## Abstract

**Introduction and importance:**

Granulomatous chronic invasive fungal rhinosinusitis (GCIFR) is a rare entity with scarce cases reported mainly in subtropical areas. Its prevalence among individuals with clinical suspicion of fungal rhinosinusitis has been reported in approximately 20% in subtropical populations, unlike North America with a prevalence of 0.5%. It is typically associated with Aspergillus flavus and the presence of noncaseating granulomas or Langerhans giant cells on histopathologic examination.

**Case presentation:**

We describe a case of a patient with clinical history of recent SARS-CoV-2 infection and development of intense cephalalgia, visual impairment, palpebral ptosis, and limitation of extraocular movements. MRI demonstrated the presence of opacification of paranasal sinuses, and a left intraconal abscess. A surgical endoscopic approach was performed and histopathologic examination revealed frontal GCIFR and maxillary fungus ball. Treatment with IV azoles provided adequate clinical response.

**Clinical discussion:**

The spectrum of the fungal rhinosinusitis disease is not clear. However, non-invasive fungal rhinosinusitis is not often found concomitantly with invasive types. GCIFR typically manifests with an indolent and gradual progression at early stages. Advanced stages can exhibit orbital and intracranial involvement leading to visual impairment, frequent relapses, and a poor prognosis. A higher incidence of invasive fungal rhinosinusitis has been reported in patients with SARS-CoV-2 infection despite an unremarkable medical history, associated with immune dysregulation.

**Conclusion:**

GCIFR is a rare condition with few cases reported in America. Because of its uncommonness, its diagnosis is often delayed leading to increased morbidity and mortality.

## Highlights

• Granulomatous chronic invasive fungal rhinosinusitis is a rare entity with scarce cases reported.
• Non-invasive fungal rhinosinusitis is not often found concomitantly with invasive types.
• A higher incidence of invasive fungal rhinosinusitis is observed in SARS-CoV-2 infection.
• Early diagnosis of fungal rhinosinusitis is essential due to possibly fatal outcome.
• Physicians should be aware of atypical manifestations for a prompt diagnosis.

## 1 Introduction

Invasive fungal rhinosinusitis (IFR) is classified into an acute and chronic types according to its time course [1]. Chronic invasive fungal rhinosinusitis (CIFR) is characterized by its long course, typically lasting for more than 12 weeks, associated by its slow onset and indolent course in early stages.

Granulomatous chronic invasive fungal rhinosinusitis (GCIFR) is a rare entity with scarce cases reported mainly in subtropical areas of Sudan, India, Saudi Arabia, and Pakistan [2]. Few cases have been observed in the United States, with approximately two reported cases of GCIFR per year [3]. It is typically associated with *Aspergillus flavus* and the presence of noncaseating granulomas or Langerhans giant cells on histopathologic examination [4]. Clinically, patients present with a chronic rhinosinusitis syndrome associated with proptosis, headache, and an enlarging mass affecting the orbit, nose and paranasal sinuses [[2], [3], [4], [5]]. However, atypical clinical manifestations should be considered. Although it manifests with an indolent and gradual progression at early stages, advanced stages can exhibit orbital and intracranial involvement leading to visual impairment, frequent relapses, and a poor prognosis [6].

We describe a case of GCIFR with orbital involvement and visual impairment associated with the presence of fungus ball in paranasal sinuses. The manuscript has been reported in line with the SCARE 2020 criteria [7].

## 2 Case Presentation

A 64-year old Mexican female patient with a history of long-standing type 2 diabetes mellitus, arterial hypertension, and dyslipidemia with adequate control presented to the emergency department with a 5-day history of progressive left blepharoedema, intense ocular pain, palpebral ptosis, and visual impairment. Upon investigation, the patient denied relevant family history and substance abuse. However, she described living in a low-resource rural community in the north of Mexico with limited health care access. PCR test for SARS-CoV-2 was performed as an institutional protocol obtaining a positive result, and the patient was given outpatient care with acetaminophen and Hypromellose eye drops without improvement. The patient concluded her 10-day isolation period at home without respiratory symptoms or complications.

The patient presented to the emergency department at our institution 35 days after onset due to low economic resources and limited family support. Upon admission referred progression of the visual impairment, horizontal extraocular movement limitation, frontal left cephalalgia, and left hemifacial paresthesia. Physical examination revealed a body temperature of 36.6 °C, blood pressure of 140/90 mmHg, a pulse of 72 beats per minute, respiratory rate of 17 breaths per minute, and blood oxygen saturation of 96% on room air.

Ophthalmologic evaluation of the left eye revealed palpebral ptosis, supraduction and adduction limitation, abduction and intorsion of the eye position, optic nerve neuropathy, orbital apex syndrome, and retinopathy associated with possible fungus vs. granulomatous process. The left eye examination showed visual acuity of 20/125 and adequate intraocular pressure without proptosis. Nasal endoscopy demonstrated no apparent necrotic or ulcerative lesions on nasal mucosa. Scarce purulent discharge was observed in superior meatus.

**Fig. 1:**
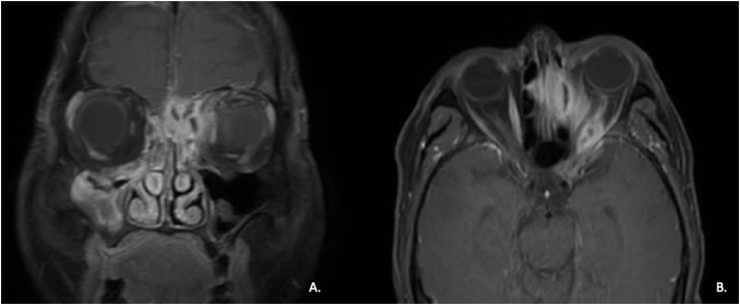
Gadolinium-enhanced T1-weighted magnetic resonance (MR) image. A, coronal view showing enhanced opacity of left frontal and ethmoidal sinuses and right maxillary sinus. B, axial view showing left ethmoidal sinus by solid mass and mucosal thickening with T1 hyperattenuation and extension to lacrimal duct and orbit, associated the presence of an intraconal abscess.

A paranasal sinus computed tomography showed soft tissue density within right maxillary sinus, opacification of frontal sinuses and left ethmoidal cells with extension and bone erosion to left cribriform plate and crista galli, ipsilateral involvement of lacrimal duct, optic nerve. A brain magnetic resonance imaging reported opacification of bilateral frontal, right maxillary sinus, and left ethmoidal sinuses by solid mass and mucosal thickening with T1 hyperattenuation and extension to left olfactory recess, cribriform plate, orbit, and lacrimal duct associated with intraconal abscess formation of approximately 0.75 cc with extension to the supraorbitary fissure and Meckel cavum compression (shown in Fig. 1).

**Fig. 2:**
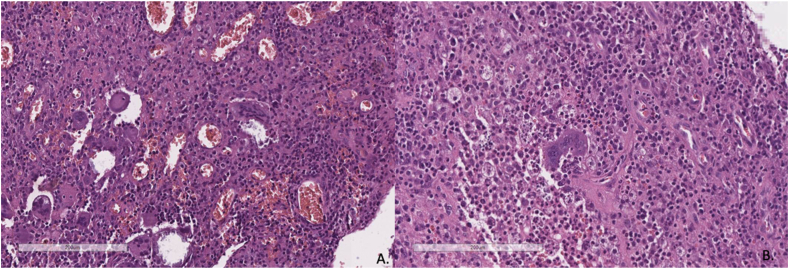
Microscopic view of histologic slides stained with hematoxylin and eosin (H&E) stain (x200). A, presence of granuloma and neoformation of blood vessels. B, presence of giant cells surrounded by lymphocytes, polymorphonuclear leukocytes, and macrophages.

On her second day after admission, a surgical endoscopic approach of the nose and paranasal sinuses with bilateral maxillary antrostomy, frontal sinus trephination, and debridement of frontal and maxillary sinus mucosa was performed by an otolaryngology specialist. Histopathologic evaluation revealed frontal chronic and acute granulomatous inflammatory process associated with mycotic structures, multinucleated giant cells and mycotic structures compatible with invasive granulomatous aspergillosis (shown in Fig. 2). Left maxillary sinus histopathology showed fungal organisms with fibrinous and necrotic exudate, with no tissue invasion or granulomatous reaction. Diagnostic challenges in our patient included her limited access to specialized health care and low economic resources, which delayed her diagnosis and treatment.

The patient began inpatient treatment with IV voriconazole for four weeks with adequate adherence and tolerability and improvement of ocular symptomatology and adequate metabolic control. No adverse events of the medication were observed. During your outpatient follow-up, frequent nasal endoscopies and ophthalmologic evaluation were performed. Ophthalmologic examination reported partial recovery of palpebral ptosis and ocular movements, without improvement in visual acuity. Control nasal endoscopic examinations showed healthy nasal mucosa with no extension or recurrence of the disease. Review of fungal microbiological cultures after four weeks isolation of Aspergillus flavus. The patient maintained outpatient treatment at an institutional hospital at her city of residence with oral itraconazole for six months. The patient attended to follow-up visits at our institution every three to six months. One-year follow-up revealed no recurrence or progression of the disease.

The patient described a painful and solitary experience, as COVID pandemic limited family access to the hospital. She maintained inpatient treatment for one month without family visits, which negatively affected her quality of hospital stay. On the other hand, she was grateful for her clinical improvement and good tolerability of the postoperative pain and medications.

## 3 Discussion

GCIFR is an uncommon disease mainly caused by *Aspergillus flavus* infection with geographic predilection towards Sudan, India, Pakistan, and Saudi Arabia, with few cases reported in North America and none reported in Mexico (Table 1) [3,8]. Its prevalence among individuals with clinical suspicion of fungal rhinosinusitis has been reported in approximately 20% in subtropical populations, unlike North America, with a prevalence of 0.5% [8,9]. This distinction is attributable to tropical climate, which gives a microaerophilic sinus environment and promotes the growth of *A. flavus* [10].

**Table 1:** Reported cases of granulomatous chronic invasive fungal rhinosinusitis in America.

Study	Year	Country	GCIFR	Isolated Fungi
			(n)	
Washburn et al. [18]	1988	North America	3	
Busaba et al. [19]	2002	North America	2	
Taxy et al. [20]	2006	North America	1	
Montone et al. [8]	2012	North America	2	Aspergillus flavus
Jariwai et al. [21]	2018	North America	1	Aspergillus flavus
Present study	2021	Mexico	1	Aspergillus flavus

GCIFR = granulomatous chronic invasive fungal rhinosinusitis.

Although non-invasive fungal rhinosinusitis is not often associated with CGIFR our patient presented both concomitant invasive and non-invasive forms of fungal rhinosinusitis. Fungus ball is a form of non-invasive fungal rhinosinusitis with an extramucosal mass of fungi associated with minimal mucosal inflammation localized unilaterally, most commonly in the maxillary sinus. Histopathologically, no granulomatous reaction or tissue invasion is seen [11].

The majority of invasive and non-invasive fungal rhinosinusitis cases are immunocompromised, aside from GCIFR which primarly affects immunocompetent individuals [12]. Although our patient had a history of diabetes mellitus with adequate control, SARS-CoV-2 infection might be a possible cause of immune dysregulation leading to exacerbation of the patient symptomatology. In Egypt and India, a higher incidence of IFR has been reported in patients with SARS-CoV-2 infection despite an unremarkable medical history [[13], [14], [15]].

Even though proptosis, enlarging mass on facial region, and nasal obstruction are main findings in advanced stages in most cases [16], our patient referred only orbital symptoms and no proptosis was seen throughout her follow-up. Severe complications of GCIFR include intracranial involvement, blindness, orbital extension, and death [3]. With orbital involvement, the orbital bone is eroded which may present as proptosis or orbital apex syndrome leading to temporal or permanent visual impairment [6]. Unfortunately, in our patient, no improvement in visual acuity despite medical and surgical management was seen.

GCIFR has a high relapse rate; however, it has a good prognosis compared with other types of IFR [6]. Surgical debridement and antifungal therapy are the mainstays of management. Voriconazole or itraconazole are the first line therapy with outcomes consistently superior to treatment with amphotericin B [17]. Rupa et al. proposed disease stages and recommended antifungal and surgical therapy. For stage 1 patients with resectable sinonasal disease and stage 2 patients with orbital or palatal extension, surgery followed by itraconazole or voriconazole is recommended. Patients with stage 3 disease with extensive disease IV followed by oral antifungal therapy are adequate [17]. Topical antifungal therapy has been introduced as an adjuvant therapy in patients with GCIFR with excellent outcomes [16]. Additionally, underlying comorbidities should be addressed and controlled with multidisciplinary consultations.

## 4 Conclusion

GCIFR is a rare condition with few cases reported in America. Because of its uncommonness, its diagnosis is often delayed leading to increased morbidity and mortality. Typical clinical manifestations include proptosis, visual changes, and sinonasal symptomatology. Nevertheless, our patient presented with atypical clinical findings including limitation of extraocular movements, palpebral ptosis, and visual impairment, without proptosis. Early diagnosis and management are essential due to its invasive and possibly fatal outcome in advanced stages. A high level of awareness of the clinician is crucial to provide and early diagnosis and management of the disease, mainly in patients with history of recent SARS-CoV-2 infection. Our patient had a delay in adequate treatment, which may have affected her visual and overall prognosis. However, debridement of paranasal sinuses and antifungal therapy with voriconazole and itraconazole provided a satisfactory control of the disease with no relapse at one-year follow-up. Management should be individualized according to the extension of the disease and medication tolerability in every patient.

## Ethical approval

The authors assert that all procedures contributing to this work comply with the ethical standards of the relevant national and institutional guidelines on human experimentation and with the Helsinki Declaration of 1975, as revised in 2008.

## Funding sources

This research did not receive any specific grant from funding agencies in the public, commercial, or not-for-profit sectors.

## Author contribution

Jose Luis Treviño-Gonzalez, substantial contribution to the conception and design, drafting of the work and revision, and final approval of the final manuscript. Karla Marisol Santos-Santillana, substantial contributions to the conception or design of the work, drafting of the work and revision, final approval of the final manuscript. Felix Maldonado-Chapa, substantial contribution to the conception of the work, final approval of the final manuscript. Josefina Alejandra Morales-del Angel, substantial contribution to the conception of the work, final approval of the final manuscript. Rosmal Cortes-Ponce, substantial contribution to the conception of the work, final approval of the final manuscript. Paola Gomez- Castillo, substantial contribution to the conception of the work, final approval of the final manuscript.

## Study approval statement

The research protocol was performed according to the Declaration of Helsinki, being approved by the local Research and Institutional Ethics Committee.

## Consent of patient

Written informed consent was obtained for publication of this case report and accompanying images. A copy of the written consent is available for review by the Editor-in-Chief of this journal on request.

## Research registration

N/a.

## Provenance and peer review

Not commissioned, externally peer-reviewed.

## Guarantor

Josefina Alejandra Morales-Del Angel MD MSc.

Otolaryngology and Head and Neck Surgery Division, School of Medicine and University Hospital “Dr. Jose E. González”, Universidad Autónoma de Nuevo León, Ave. Madero y Gonzalitos s/n, Mitras Centro, 64460, Monterrey, Nuevo Leon, Mexico.

E-mail: jamoralesorl@gmail.com.

Fax Number: 8183891111.

## Declaration of competing interest

Authors declare no conflicts of interest.
